# Supplementary material for: Predicting Effective Diffusivity of Porous Media from Images by Deep Learning
Source: Sci Rep. 2019 Dec 31;9:20387. doi: 10.1038/s41598-019-56309-x (PMC6938523; doi:10.1038/s41598-019-56309-x)
Supplement: Supplementary file 1 — Supplementary Information [file 41598_2019_56309_MOESM1_ESM.pdf]

Predicting Effective Diffusivity of Porous Media from Images by Deep Learning

Haiyi Wu,[1] Wen-Zhen Fang,[1,2] Qinjun Kang,[3,*] Wen-Quan Tao,[2,*] and Rui Qiao[1,*]

[1] Department of Mechanical Engineering, Virginia Tech, Blacksburg, VA 24061, USA

[2] Key Laboratory of Thermo-Fluid Science and Engineering, MOE, Xi'an Jiaotong University, Xi'an, China

[3] Earth and Environmental Sciences Division, Los Alamos National Laboratory, Los Alamos, NM 87545, USA

1. Basic concept of neural network and convolutional neural network

A classical neural network consists of N layers with the kth layer containing n_k neurons (see Fig. S1-a for an example), A neuron i in a layer k processes the output of layer $k-1$ neurons (x_j^{k-1}, $j = 1 \cdots n_{k-1}$) by first assigning weights and biases to this output and then passing the result to a nonlinear activation function f to generate its output

$$x_i^k = f(\sum_{j=1}^{n_{k-1}} W_{i,j}^k x_j^{k-1} + B_i^k) \quad \text{(S1)}$$

where $W_{i,j}^k$ and B_i^k are the weight and bias of neuron i for the output of the neuron j in the layer $k-1$. Equation (S1) can be written compactly as

$$\mathrm{X}^k = f(W^k \mathrm{X}^{k-1} + B^k) \quad \text{(S2)}$$

where $\mathrm{X}^{\mathrm{k-1}}$ is a $n_{k-1} \times 1$ vector, W^k is a $n_k \times n_{k-1}$ matrix, and B^k is a $n_k \times 1$ vector. A neuron network is defined once the number of neuron layers (N), the number of neurons in each layer (n_k), the weight and bias of all neurons (W^k and B^k, $k = 1, 2, \cdots, N$), and the activation function (f) are determined. Of these parameters, N, $n_{1,2,\cdots,N}$, and f are termed *hyperparameters* and are specified when developing a neural network. W^k and B^k are termed *learnable parameters* to be determined during the training of the neural network. Neural networks can be used for tasks such as classifying images. Because each neuron in an arbitrary layer k is fully connected with all neurons in layer $k-1$ and $k+1$ and the

* Correspondence and requests for materials should be addressed to R.Q. (ruiqiao@vt.edu), Q.K. (qkang@lanl.gov), and W.-Q.T. (wqtao@mail.xjtu.edu.cn).

learnable parameters W^k and B^k are by default different for each neuron layer, the number of learnable parameters is enormous when the input of a neural network features massive data (e.g., when an input image has a large number of pixels and multiple color channels). Hence, classical neural networks can involve prohibitive computational cost and is prone for overfitting.

The convolutional neural network (CNN) is a variant of the classical neural network. As shown in Fig. S1-b, a CNN typically consists of three types of layers: the convolutional layer, the pooling layer, and the fully-connected layer. Each type of layers can be included in any number and sequence. The fully-connected layers in CNN are identical to those in the classical neural network and we only outline the first two types of layers.

A convolutional layer contains a 3D volume of neurons arranged in the width, height, and depth directions (see Fig. S1-b). Both the neurons of layer k and their output are often termed volume k. Even though the neurons in a convolutional layer process information in a similar way as depicted in Equation (S2), they differ from those in the classical neural networks in that (1) each neuron in a volume k only processes the output from a small number of neurons in volume $k-1$ and (2) within each depth slice of a volume k, all neurons have the same weights and biases. Due to these differences, the number of learnable parameters in CNN is greatly reduced compared to that in classical neural networks.[1,2]

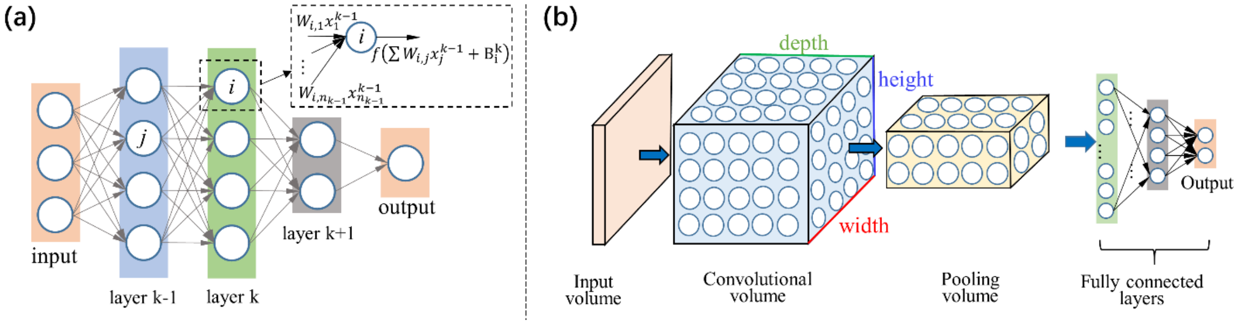

Figure S1. The schematics of a simple classical neural network (a) and a simple convolutional neural network (b).

To further reduce the number of learnable parameters and overfitting, pooling layers are usually inserted periodically between convolutional layers. A pooling layer essentially downsamples the output of the convolutional layer preceding it and progressively reduces the neuron volume.[3] A typical pooling layer

operates independently on each slice of the output volume of its preceding convolutional layer volume using algorithms such as the max pooling, average pooling, or L_2 norm pooling.[4]

2. Computational framework for predicting diffusivity of porous media

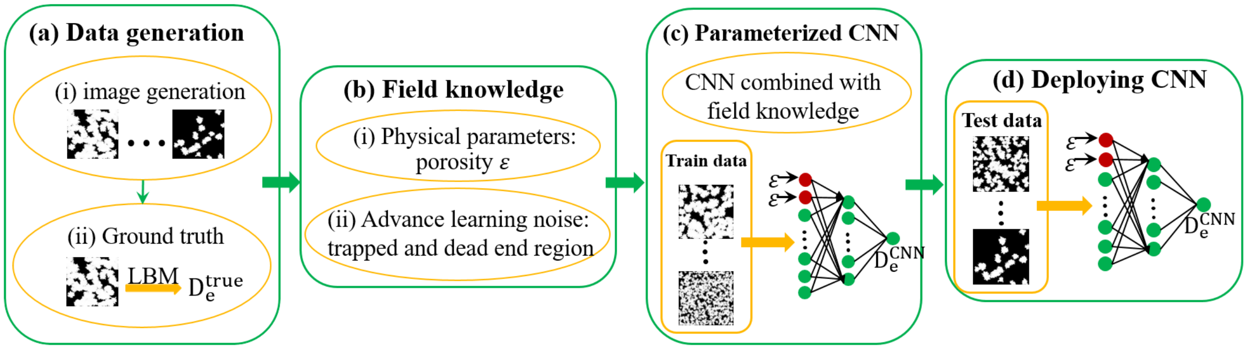

Figure S2. Overview of the computational framework of using CNN to predict the effective diffusivity of porous media from their images.

3. ResNet model for predicting the diffusivity of porous media

In addition to CNN model described in Fig. 2 of the main text, we also tested the deep residual network, which often gives better accuracy than the CNN in the main text. Deep residual networks are stacked with many residual learning blocks, which contain several convolutional layers and a short cut connection between the input and the output before the activation layer. Deep residual networks are substantially deeper than the regular CNN model, are easier to optimize, and can gain considerable accuracy from the increased layers. Training very deep residual networks, however, involves much higher computation cost than the CNN described in the main text.

Here, we implemented the Resnet50 model[5] to predict the effective diffusivity of porous media using the datasets in our original manuscript, both with and without removing the trapped regions in the preprocessing step. The architecture of the Resnet50 model is same as that in Ref. [5]. The training model is converged through minimizing the loss function Eq. 7 by the Adam Optimizer[6] (learning rate $\gamma = 10^{-4}$). The learning rate is reduced by 50% every 300 epochs to ensure stable convergence and the training model stops at 1600 epochs. The average mean square error (MSE), mean truncated relative error (MTRE) and the Pearson R^2 scores are summarized in the Table below.

The results of the Resnet50 model are summarized in Table S1 and Fig. S3. We observe that, compared to the model in the main text, the Resnet50 model perform slightly better: the MSE is reduced by ~20% for dataset with and without the preprocessing step. The improvement of the MTRE is smaller: ~8% and ~3% for dataset without and with preprocessing, respectively. Although the prediction accuracy for Resnet50 is slightly higher than the CNN model in the main text, the Resnet50 requires more computational resources to train. For example, compared to the CNN model in the main text, the Resnet50 model involves 10 times more parameters and requires 5 times longer training time.

Table S1. comparison between the results by CNN models and resnet50 model

	Trained using the loss function Equ. 7 with original data			Trained using the loss function Equ. 7 with preprocessed image as input		
	MSE	MTRE	R^2	MSE	MTRE	R^2
CNN	8.64×10^{-4}	68.8%	0.9903	$6.92\times10^{-}$	29.7%	0.9912
Resnet50	7.26×10^{-4}	60.50%	0.9918	$5.34\times10^{-}$	26.56%	0.9932

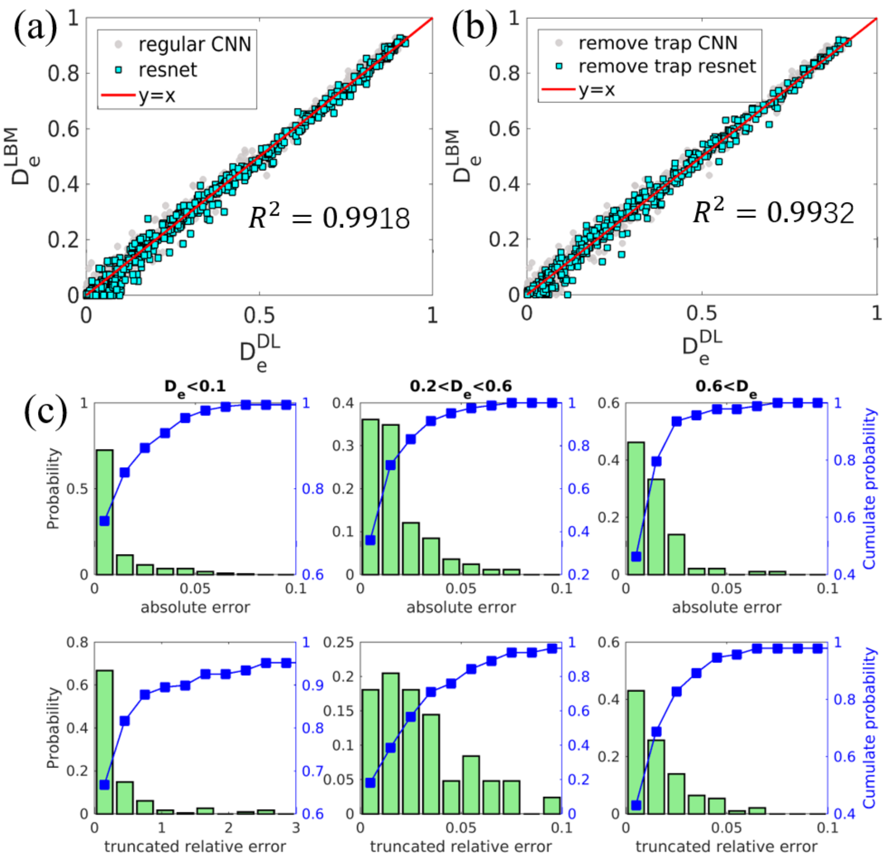

Figure S3. (**a**) The effective diffusivity predicted by the Resnet50 model trained using a loss function based on the mean square error (Equation 7) with the dataset without preprocessing. (**b**) The effective diffusivity predicted by the Resnet50 model trained using a loss function based on the mean square error (Equation 7) with the dataset preprocessed by removing the trapped regions. (**c**) Distribution of the absolute error (top panels) and truncated relative error (lower panels) of the predictions of the Resnet50 model (trained with dataset that removed the trapped regions) for the porous structures in the testing dataset with different D_e.

References

1 Kalchbrenner, N., Grefenstette, E. & Blunsom, P. A convolutional neural network for modelling sentences. *arXiv preprint arXiv:1404.2188* (2014).

2 Krizhevsky, A., Sutskever, I. & Hinton, G. E. in *Advances in neural information processing systems.* 1097-1105.

3 Tolias, G., Sicre, R. & Jégou, H. Particular object retrieval with integral max-pooling of CNN activations. *arXiv preprint arXiv:1511.05879* (2015).

4 Zeiler, M. D. & Fergus, R. Stochastic pooling for regularization of deep convolutional neural networks. *arXiv preprint arXiv:1301.3557* (2013).

5 He, K., Zhang, X., Ren, S. & Sun, J. in *European conference on computer vision.* 630-645 (Springer).

6 Kingma, D. P. & Ba, J. Adam: A method for stochastic optimization. *arXiv preprint arXiv:1412.6980* (2014).
